# Impact of Routine and Selective Preoperative Endoscopic Retrograde Cholangiopancreatography with Stent Placement on Postoperative and Oncologic Outcomes Following Pancreaticoduodenectomy for Pancreatic Ductal Adenocarcinoma

**DOI:** 10.3390/biomedicines13020333

**Published:** 2025-02-01

**Authors:** Pauline Aeschbacher, Anna Silvia Wenning, Shadi Katou, Haluk Morgul, Mazen Juratli, Felix Becker, Ibrahim Büdeyri, Beat Gloor, Andreas Pascher, Benjamin Struecker, Andreas Andreou

**Affiliations:** 1 Department of Visceral Surgery und Medicine, lnselspital, Bern University Hospital, University of Bern, 3010 Bern, Switzerland; 2 Department of General, Visceral and Transplant Surgery, University Hospital Munster, 48149 Münster, Germany

**Keywords:** preoperative biliary stenting, pancreatic adenocarcinoma, postoperative morbidity, overall survival

## Abstract

**Background:** According to current guidelines, preoperative endoscopic retrograde cholangiopancreatography (ERCP) with biliary stenting (ERCP/stenting) is often necessary in patients with obstructive jaundice due to pancreatic ductal adenocarcinoma (PDAC), including severe jaundice (bilirubin > 250 umol/l), pruritus, cholangitis, cholestatic liver dysfunction, renal failure, severe malnutrition, or delayed surgery for tumors requiring neoadjuvant chemotherapy. We aimed to investigate the impact of preoperative ERCP/stenting on postoperative and long-term outcomes following pancreaticoduodenectomy (PD) for PDAC. **Methods:** Clinicopathological data of patients who underwent partial/total PD for PDAC between 2012 and 2019 in two hepato-pancreato-biliary centers in Germany and Switzerland were assessed. We compared patients treated with preoperative ERCP/stenting with those directly undergoing surgery according to postoperative morbidity, postoperative mortality, overall survival (OS) and disease-free survival (DFS). **Results:** During the study period, 192 patients underwent partial/total PD for PDAC. ERCP/stenting was performed in 105 patients, and 87 patients underwent resection without prior intervention. Postoperative 90-day overall morbidity rate (71% vs. 56%, *p* = 0.029) and superficial surgical site infection (SSI) rate (39% vs. 17%, *p* < 0.001) were significantly worse following preoperative ERCP/stenting. Major postoperative morbidity rate (18% vs. 21%, *p* = 0.650), organ/space SSI rate (7% vs. 14%, *p* = 0.100), and 90-day postoperative mortality rate (4% vs. 2%, *p* = 0.549) did not significantly differ between the two groups. After excluding 44 patients for whom the indication for ERCP/stenting was not consistent with current guidelines, ERCP/stenting was associated with a higher superficial SSI rate (36% vs. 17%, *p* = 0.009) and shorter length of stay (12 vs. 16 days, *p* = 0.004). Median OS (ERCP/stenting: 18 months vs. no ERCP/stenting: 23 months, *p* = 0.490) and median DFS (ERCP/stenting: 14 months vs. no ERCP/stenting: 18 months, *p* = 0.645) were independent from the utilization of ERCP/stenting. **Conclusions:** Preoperative ERCP/stenting in patients with PDAC can be performed without increasing organ/space SSI, major perioperative morbidity, and mortality rates and without worsening oncologic outcomes. However, it is associated with higher superficial SSI rates. If ERCP/stenting is not performed routinely but according to current guidelines, it is also associated with a shorter length of hospital stay. Further refinement of the indications for preoperative ERCP/stenting may reduce superficial SSI rates.

## 1. Introduction

Pancreatic ductal adenocarcinoma (PDAC) has an incidence of 8.1/100’000 in Europe, and it is the third leading cause of cancer-related death after lung and colorectal cancer. PDAC has the poorest prognosis of all solid tumors, with a 5-year overall survival (OS) rate of less than 5% [1]. Pancreatic cancer is often referred to as the “silent killer”, as patients typically remain oligosymptomatic until an advanced stage [2]. Unfortunately, approximately 50% of patients present with metastasis at the time of diagnosis and only 20% are eligible for upfront resection [3]. The evolution of multimodal treatment, both adjuvant and neoadjuvant, offered better results for surgically treated PDAC patients, with a 5-year OS rate increase from 1.5% to 17.4% between 1975 and 2011 [1]. In patients with combined surgical and oncological treatment, the 5-year OS rate has increased and is currently up to 40% [4,5,6].

Painless jaundice is a common symptom of patients with a pancreatic head tumor which occurs when the tumor invades or compresses the common bile duct. According to data from the German Pancreatic Surgery Registry, preoperative symptoms are common in these patients. In an analysis of 2643 patients, 2380 (90%) presented with one or more symptoms, including jaundice (40%) and/or biliary obstruction treated with a biliary stent (41%) being the most frequent [7]. Jaundice has a negative impact on the renal, hepatic, immunological, nutritional, and coagulation status and, therefore, represents an additional challenge in patients’ management [8]. Biliary stenting is often mandatory in case of symptomatic or severe jaundice [9].

The preferred procedure to perform biliary decompression is an endoscopic retrograde cholangiopancreatography with biliary stent placement (ERCP/stenting) [10]. In the past, ERCP/stenting has been routinely performed prior to oncological pancreaticoduodenectomy (PD) [11]. However, in many studies, ERCP/stenting was associated with increased postoperative complications, including wound infections, and a systematic review of 32 studies concluded that avoiding preoperative ERCP/stenting, if possible, may be the preferred option for jaundiced patients with resectable PDAC [12]. On the contrary, in a meta-analysis published in 2016 that included patients with malignant biliary jaundice requiring surgery, ERCP/stenting resulted in significantly fewer major adverse effects [13]. In addition, preoperative treatment of biliary obstruction, and, thus, a decrease in cholestasis, may allow improvement of patients’ general and nutritional status, as well as renal and hepatic function, thereby improving patients’ performance status and leading to better postoperative outcomes [8].

Taken together, biliary decompression is currently not recommended as a routine procedure in asymptomatic patients with bilirubin levels <250 µmol/L. It is usually performed in cases of severe jaundice, pruritus, cholangitis, cholestatic liver dysfunction, renal failure, severe malnutrition, or delayed surgery for tumors requiring neoadjuvant chemotherapy [9,10]. The decision to perform preoperative biliary decompression is usually made on an individual basis by the treating gastroenterologist or surgeon.

The impact of ERCP/stenting on outcomes following pancreatic resection has been mostly investigated when biliary drainage was performed on a routine basis prior to resection and not when restricted to current indications. In our retrospective study, we aimed to evaluate the impact of preoperative ERCP with stent placement on postoperative and long-term outcomes after curative PD for PDAC in two hepato-pancreato-biliary centers with a focus on routine ERCP/stenting vs. stent placement restricted to indications for biliary drainage according to current guidelines [9].

## 2. Material and Methods

### 2.1. Patient Inclusion Criteria

All consecutive patients who underwent partial/total PD for PDAC of the pancreas head or body between 2012 and 2019 at two hepato-pancreato-biliary centers in Germany (University Hospital of Munster) and Switzerland (lnselspital, Bern University Hospital) were included in the study. Patients treated with a percutaneous transhepatic biliary drainage or endoscopic ultrasound-guided hepaticogastrostomy (EUS-HGS), and patients < 18 years old, were excluded from the analysis. Patient data collection and analysis from the two cohorts in Switzerland and Germany were approved by the local ethical committees in Bern (2019-01171) and Munster (ID 2019-636-f-S), respectively.

### 2.2. Preoperative Management

Patients with PDAC were systematically clinically evaluated, and tumor staging was performed using computer tomography with biphasic contrast agent, magnetic resonance imaging, and endoscopic ultrasound examination. All patients were discussed at the local multidisciplinary tumor boards which recommended primary resection for resectable tumors and preoperative chemotherapy for borderline resectable or locally-advanced PDAC [14].

The decision to proceed with a biliary decompression for patients initially presented in both centers was made individually according to the severity of the cholestasis and its associated complications, the general and nutritional status of the patient, and the estimated delay to surgery according to current guidelines [9]. Usually, plastic stents have been used for biliary decompression prior to resection, and metallic stents have been reserved for palliative situations.

### 2.3. Surgical Procedure

The oncologic procedures in our study included total or partial PD depending on the location of the tumor, the quality of the remaining pancreas, and the risk of postoperative pancreatic fistula. The resections were performed by open or laparoscopy-assisted procedure as previously reported [15]. Drains were always placed at the end of the operation next to the hepaticojejunostomy and the pancreatic anastomosis to monitor for anastomotic leak. If necessary, resection and reconstruction of the portal vein or superior mesenteric vein were performed either by direct anastomosis or using a graft.

### 2.4. Postoperative Management

Postoperatively, patients were usually admitted to an intermediate or intensive care unit before being transferred to the surgical ward. They were monitored for postoperative complications such as anastomotic leak, postoperative pancreatic fistula (POPF), postpancreatectomy hemorrhage (PPH), or surgical site infection (SSI). POPF and PPH were diagnosed and classified according to definitions of the International Study Group on Pancreatic Surgery (ISGPS) [16,17]. Postoperative bile leak was classified according to definitions of the International Study Group for Liver Surgery (ISGLS) [18]. Postoperative morbidity was classified using the Clavien–Dindo classification [19]. Postoperative complications requiring intervention, reoperation, or readmission to intensive care unit were considered major complications (Clavien–Dindo grade ≥ 3a) [19]. Postoperative mortality was defined as any death within 90 days after surgery.

### 2.5. Statistical Analysis

The primary endpoint of this study was major postoperative morbidity. Secondary endpoints were overall postoperative morbidity and mortality, as well as disease-free survival (DFS) and OS. Therefore, postoperative complications of patients who underwent preoperative ERCP/stenting were compared with those of patients without ERCP/stenting prior to the oncologic PD. Furthermore, a second analysis was performed to compare the subgroup of patients with a strict indication for ERCP/stenting (i.e., severe jaundice with bilirubin > 250 umol/l, pruritus, cholangitis, cholestatic liver dysfunction, renal failure, severe malnutrition, and need for neoadjuvant chemotherapy [9]) with the subgroup of patients without ERCP/stenting. Quantitative and qualitative variables were expressed as medians (range) and frequencies, respectively. The chi-square or Fisher’s exact test and the Mann–Whitney *U* test were used to compare categorical and continuous variables, as appropriate. OS and DFS were calculated from the date of surgical procedure to the date of death or recurrence, respectively, or the last follow-up using the Kaplan–Meier method. Log-rank test was used to compare OS and DFS between patients with or without ERCP/stenting prior to PD. *p* values < 0.05 were considered statistically significant. For statistical analysis, SPSS software package, version 25 by IBM (Armonk, NY, USA) was used.

## 3. Results

### 3.1. Patient Characteristics

During the study period, 192 patients were included in our study. ERCP/stenting was performed in 105 patients, and 87 patients underwent resection without prior intervention. Clinicopathologic characteristics of the 192 study patients are presented in Table 1. Median time from ERCP/stenting to surgery was 16 days (range 4–130) without neoadjuvant chemotherapy and 141 days (range 90–270) in case of neoadjuvant chemotherapy. Post-ERCP complications were reported in 20 patients (19%). The most frequent complication was stent obstruction (n = 13, 12.4%), followed by post-ERCP pancreatitis (n = 6, 5.7%, out of which two were severe), and bleeding (n = 1, 1.0%). In most cases, bilirubin levels were significantly reduced following drainage. Only five cases showed an improvement of less than 50 µmol/L, with levels still higher than 20 µmol/L. In three of these cases, the available bilirubin levels were measured only one day after drainage, and further reductions are likely to have occurred during subsequent outpatient measurements. Only two patients did not experience a significant improvement in hyperbilirubinemia, despite a functioning stent, potentially reflecting liver damage caused by prolonged cholestasis.

### 3.2. Comparison of Clinicopathological Characteristics and Postoperative Outcomes According to the Utilization of ERCP/Stenting

The comparison of patient characteristics and postoperative outcomes according to the utilization of preoperative ERCP/stenting is summarized in Table 1. Patients with preoperative ERCP/stenting had a higher maximum bilirubin level prior to the intervention compared to patients who directly underwent surgery (*p* < 0.001). No other differences were found between the two groups regarding preoperative characteristics.

Total PD was more frequently performed in patients without preoperative ERCP/stenting (36% vs. 20%, *p* = 0.015) due to PDAC of the body of the pancreas causing no jaundice. Intraoperative bile sampling for microbiology was routinely performed in patients with a biliary stent (92%) but only occasionally in patients without a stent (23%). Importantly, 99% of patients in the stent group and 35% of patients in the no-stent group had positive microbiological findings (*p* < 0.001).

Tumor stage and grading were similar in both groups except for venous invasion, which was more frequent in patients without preoperative ERCP/stenting (66% vs. 79%, *p* = 0.037).

The rate of 90-day postoperative overall morbidity was higher in patients who underwent preoperative ERCP/stenting (71% vs. 56%, *p* 0.029); however, no difference was observed in 90-day major postoperative morbidity (18% vs. 21%, *p* = 0.650) or 90-day postoperative mortality (4% vs. 2%, *p* = 0.549) between the two groups. Among all reported complications, only superficial SSI was significantly higher in patients with preoperative stenting (39% vs. 17%, *p* < 0.001). No statistically significant differences were found for organ/space SSI, POPF, and PPH between the two groups.

### 3.3. Comparison of Clinicopathological Characteristics and Postoperative Outcomes According to the Utilization of ERCP/Stenting Based on the Presence of Severe/Symptomatic Jaundice or the Need for Neoadjuvant Chemotherapy

The indication for preoperative biliary drainage was severe or symptomatic jaundice in 48 (46%) patients and neoadjuvant treatment in 13 (13%) patients. Forty-four (42%) patients for whom the indication for ERCP/stenting could not be clarified by analyzing patients’ medical record were excluded. These ERCP/stenting procedures were often performed in a referring hospital (66%) prior to admission to our centers or during investigation for unclear cholestasis (4%).

In the subgroup analysis of patients with severe or symptomatic jaundice or those administered neoadjuvant chemotherapy who underwent ERCP/stenting, postoperative morbidity, major morbidity, and mortality were similar compared to patients who were not treated with ERCP/stenting (71% vs. 56%, *p* = 0.08; 18% vs. 21%, *p* = 0.689; 7% vs. 2%, *p* = 0.196, respectively). Among all reported complications, only superficial SSI was significantly higher in patients with preoperative ERCP/stenting (36% vs. 17%, *p* = 0.009). The length of hospital stay was shorter after preoperative ERCP/stenting (12 vs. 16 days, *p* = 0.004), (Table 2).

### 3.4. Comparison of Oncologic Outcomes According to the Utilization of ERCP/Stenting

**Figure 1:**
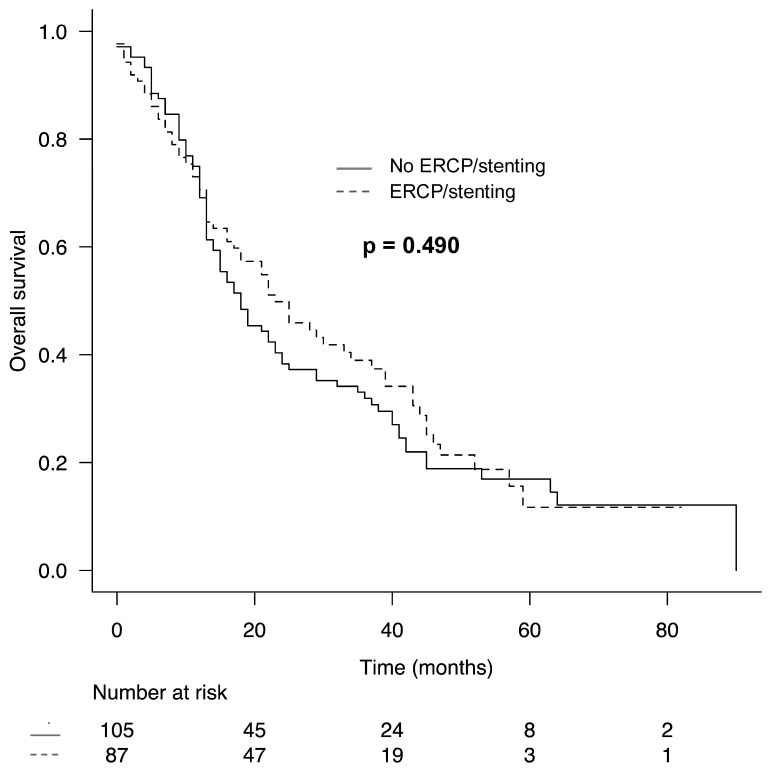
Comparison of overall survival in patients undergoing partial/total pancreaticoduodenectomy for ductal adenocarcinoma of the pancreas according to utilization of ERCP/stenting.

**Figure 2:**
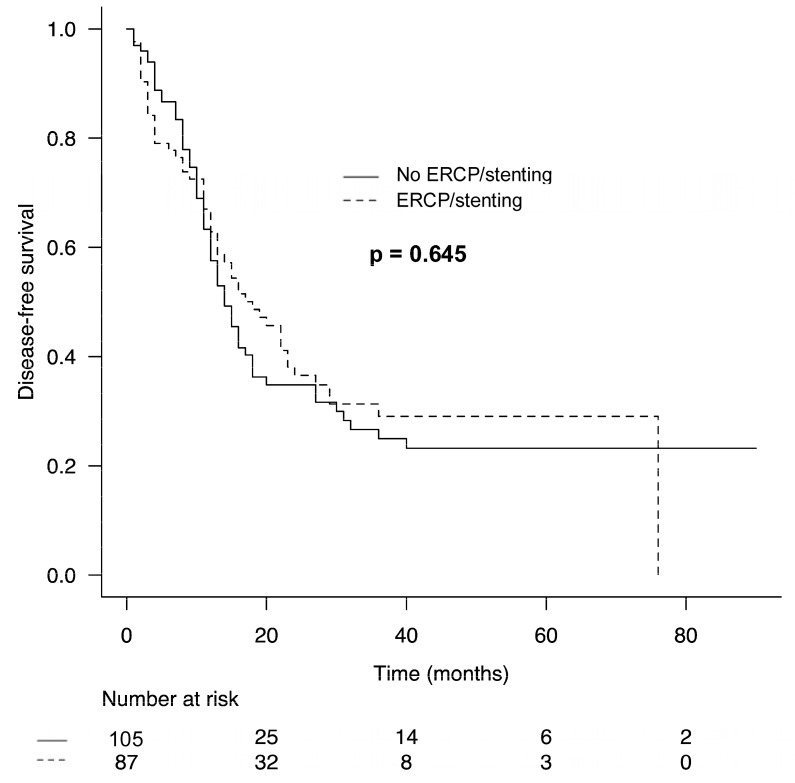
Comparison of disease-free survival in patients undergoing partial/total pancreaticoduodenectomy for ductal adenocarcinoma of the pancreas according to utilization of ERCP/stenting.

Median OS (ERCP/stenting: 18 months vs. no ERCP/stenting: 23 months, *p* = 0.490) and median DFS (ERCP/stenting: 14 months vs. no ERCP/stenting: 18 months, *p* = 0.645) were not associated with the utilization of preoperative ERCP/stenting (Figure 1 and Figure 2). In the subgroup analysis of patients with severe or symptomatic jaundice or those who were administered neoadjuvant chemotherapy and underwent ERCP/stenting, median OS and DFS were also comparable to those of patients who were not treated with ERCP/stenting (OS: ERCP/stenting: 17 months vs. no ERCP/stenting: 23 months, *p* = 0.180 and DFS: ERCP/stenting: 13 months vs. no ERCP/stenting: 18 months, *p* = 0.132).

## 4. Discussion

Preoperative biliary stenting in patients planned for PD has been extensively studied, and several studies and meta-analyses presented valid evidence against routine ERCP/stenting prior to surgery [12,20,21]. In accordance with these data, we also found a higher postoperative morbidity rate (71% vs. 56%, *p* = 0.029) following ERCP/stenting, mainly because of the higher rates of superficial SSI (39% vs. 17%, *p* < 0.001). However, no statistical differences were observed for major postoperative morbidity, organ/space SSI, POPF, PPH, and postoperative mortality. Median OS and median DFS were also not associated with utilization of preoperative ERCP/stenting. In the subgroup analysis of patients who underwent ERCP/stenting due to severe or symptomatic jaundice or the need for neoadjuvant chemotherapy, preoperative ERCP/stenting was only associated with higher superficial SSI rates (22% vs. 17%, *p* = 0.009) and, interestingly, shorter lengths of hospital stay (12 vs. 16 days, *p* = 0.004) compared to patients without ERCP/stenting prior to surgery.

Despite ample data documenting adverse effects of ERCP/stenting, there are still too many interventions performed as a routine procedure prior to PD (42% in our study). This was even worse in a recent analysis of data from the German DGAV StuDoQ/Pancreas registry. Among 480 patients receiving ERCP/stenting in this cohort, only a minority of 33% had a history of jaundice [11].

Further data on the correct indication for preoperative ERCP/stenting is lacking, as most of the available data are based on patients having drainage with bilirubin levels lower than 250 µmol/L. No data are available on the benefits and risks of ERCP/stenting in a population with severe (bilirubin levels higher than > 250 µmol/L) or symptomatic (cholangitis, pruritus) cholestasis. Interestingly, in our study, postoperative morbidity was comparable in patients with or without preoperative ERCP/stenting when the drainage indication reflected the current guidelines [9]. However, superficial SSI was still higher in patients with preoperative ERCP/stenting, most likely due to the colonized biliary tree and the consequent contaminated operative field.

Oehme et al. suggested that the time period from stenting to surgery is also a relevant aspect which may influence postoperative outcome [22]. They reported a lower rate of postoperative complications when pancreatic resection was performed within four weeks after ERCP/stenting. In our study, the majority of patients (68%) underwent pancreatic resection within 4 weeks after ERCP/stenting (patients with neoadjuvant chemotherapy excluded). We did not identify a difference in terms of overall and major postoperative morbidity between patients who underwent resection before or after 4 weeks.

In our subgroup analysis of patients with severe or symptomatic jaundice, ERCP/stenting was associated with a shorter length of hospital stay (12 vs. 16 days, *p* = 0.004). This difference may be associated with the lower rate of total PD performed in the ERCP/stenting group, given that total PD requires in-depth introduction and regulation of diabetes Typ 3c [23]. Moreover, severe prolonged jaundice is associated with impaired liver function, and previous studies have indicated that postoperative recovery after hepatobiliary surgery may be quicker if biliary decompression has been preoperatively performed [24].

Patients with cholestasis and planned neoadjuvant treatment should receive biliary drainage to avoid the risk of developing cholestasis-related complications, such as cholangitis, and thus compromising the ongoing oncologic treatment. A better assessment of the indications for biliary stenting in this specific setting is necessary. A recent retrospective study reported higher postoperative infectious complications and SSIs for patients who received neoadjuvant chemotherapy and biliary stenting prior to pancreatic resection, but patients’ bilirubin levels were not reported, and it is unknown whether this study included patients with severe jaundice [25]. In our study, only 4 out of 13 patients who received neoadjuvant chemotherapy underwent a pancreatic resection without preoperative ERCP/stenting. However, these four patients did not have cholestasis.

In accordance with existing data, we observed a higher rate of superficial SSI in patients with preoperative ERCP/stenting compared to those without ERCP/stenting in our study (*p* < 0.001) [26]. Elliott et al. published a recent analysis based on the National Surgical Quality Improvement Program–Hepatopancreaticobiliary Collaborative database which identified risk factors for superficial and organ/space SSI after pancreatic surgery. Their data showed that preoperative biliary drainage is a risk factor for superficial SSI but not for organ/space SSI, which is more related to postoperative POPF than to biliary drainage [26]. Therefore, the authors highlight the importance of a distinction between superficial and organ/space SSI, which can otherwise result in misleading conclusions.

Even if superficial SSI might have a relatively low impact on the postoperative outcome, it is a major factor resulting in a longer length of hospital stay and relevant costs [27]. Consequently, several prophylactic measures are currently being investigated to prevent the higher risk for superficial SSI in hepato-pancreato-biliary surgery after ERCP/stenting, such as antibiotics or intraoperative wound irrigation [26,27]. However, evidence on this subject remains low, and SSI remains a relevant issue after pancreatic resections. Intraoperative bile microbiology was performed more often in our patients with a biliary stent, as previously reported [28]. These patients also had a significantly higher rate of positive microbiological findings than those without preoperative ERCP/stenting when microbiology was performed. The higher number of SSIs is most likely explained by these findings, as reported in other studies [22,26]. This finding may result in the recommendation for perioperative antibiotic prophylaxis for stent-related bacteriobilia [10,28,29]. However, the benefit of systematic postoperative antibiotic administration is limited by the risk of promoting antimicrobial resistance and Clostridium difficile colitis. According to a recent multicenter randomized study, piperacillin-tazobactam seems to be the most suitable antibiotic regimen for perioperative prophylaxis in patients undergoing PD, as it reduces postoperative SSI, pancreatic fistula, and postoperative sepsis [30]. Nevertheless, patients undergoing preoperative ERCP/stenting should be closely monitored for early signs of infection. These complications must be anticipated with appropriate treatment (e.g., drainage, wound care) and, if necessary, antibiotic treatment adapted to the intraoperative microbiology [9].

Additionally, ERCP itself carries inherent risks of complications that can disrupt surgical timing, feasibility, and outcomes. Although techniques have been developed to optimize ERCP success and minimize post-ERCP complications, biliary cannulation remains a challenging procedure [31]. Reported complication rates after ERCP vary from 6% up to 26% and generally consist of cholangitis, obstruction, pancreatitis, perforation, or bleeding [10,28]. The most feared complication is the post-ERCP pancreatitis, which may delay or even compromise pancreatic resection and negatively impact the oncologic outcome of patients with PDAC [32]. Furthermore, post-ERCP complications might have an indirect impact on surgical outcomes, biliary colonization being potentially responsible for higher postoperative infections or post-ERCP pancreatitis being responsible for more challenging and longer surgeries. On the other hand, ERCP has, in addition to decompression of the main bile duct, the advantage of providing a diagnostic tool together with the simultaneous use of endosonography and fine-needle aspiration. Therefore, the decision to perform ERCP should be evaluated for each patient individually.

Increased postoperative complications following ERCP/stenting may delay adjuvant chemotherapy and thus also have a negative impact on OS and DFS. Previous studies have shown that complications following pancreatic resection are significantly associated with worse oncologic outcome [33,34]. However, OS and DFS were also not associated with the use of preoperative ERCP/stenting in our study.

Our study has several limitations inherent to a retrospective design. Some heterogeneity between patients with or without preoperative ERCP/stenting (resection type and vascular resection) might also be a confounding factor. However, our data included a relatively high number of patients from two different hepato-pancreato-biliary centers, where ERCP/stenting was performed according to current recommendations. To the best of our knowledge, this is the first study to evaluate the outcomes after PD for PDAC taking into consideration the current indications for the utilization of preoperative ERCP/stenting.

## 5. Conclusions

Preoperative ERCP/stenting for patients with PDAC can be performed without increasing organ/space SSI, major postoperative morbidity, or mortality rates. It also does not have a negative impact on oncologic outcomes. However, it is associated with higher superficial SSI rates. If ERCP/stenting is not performed routinely but only for patients with severe and symptomatic jaundice or with a need for neoadjuvant chemotherapy, it is also associated with a shorter length of hospital stay. Therefore, the decision to perform ERCP/stenting should be individually made in every case, evaluating the benefit–risk ratio according to current guidelines. Further refinement of the indication for preoperative ERCP/stenting may reduce superficial SSI rates.

## Figures and Tables

**Table 1:** Comparison of clinicopathological characteristics of 192 patients undergoing partial/total pancreaticoduodenectomy for ductal adenocarcinoma of the pancreas according to utilization of ERCP/stenting.

Variable	ERCP/Stenting (n = 105)	No ERCP/Stenting (n = 87)	All Patients (n = 192)	*p* Value
Gender, n (%)				0.377
Female	55 (52)	40 (46)	95 (49)	
Male	50 (48)	47 (54)	97 (51)	
Age median (range)	69 (37–84)	67 (39–84)	68 (37–84)	0.999
Heart disease, n (%)	55 (52)	54 (62)	109 (57)	0.177
Kidney disease, n (%)	16 (15)	12 (14)	28 (15)	0.778
Pulmonary disease, n (%)	19 (18)	18 (21)	37 (19)	0.650
Liver disease, n (%)	10 (10)	5 (6)	15 (8)	0.332
Neurologic disease, n (%)	8 (8)	11 (13)	19 (10)	0.246
Diabetes, n (%)	29 (28)	27 (31)	56 (29)	0.604
BMI, kg/m^2^, median (range)	24 (16–46)	25 (16–43)	25 (16–46)	0.899
BMI > 30, n (%)	14 (13)	9 (10)	23 (12)	0.526
Smoking, n (%)	26 (25)	31(36)	57 (30)	0.101
Alcohol consumption, n (%)	21 (20)	26 (30)	47 (25)	0.113
ASA status, n (%)				0.449
1	1 (1)	0 (0)	1 (1)	
2	37 (35)	24 (28)	61 (32)	
3	59 (56)	53 (61)	112 (58)	
4	8 (8)	10 (11)	18 (9)	
CA 19-9 preoperative, median (range)	226 (1–23539)	256 (2–63690)	235 (1–63690)	0.744
Maximum level of bilirubin, µmol/L, median (range)	153 (2–592)	8 (2–646)	79 (2–646)	<0.001
Difference of bilirubin level before and after drainage, µmol/L, median (range)	93.5 (2–361)			
Intraoperative bile microbiology performed, n (%)	97 (92)	20 (23)	117 (61)	
Positive intraoperative bile microbiology, n (%)	96 (99)	7 (35)	103 (88)	<0.001
Resection type, n (%)				0.015
Partial pancreaticoduodenectomy	84 (80)	56 (64)	140 (73)	
Total pancreaticoduodenectomy	21 (20)	31 (36)	52 (27)	
Operating time, h, median (range)	6 (4–11)	6 (4–11)	6 (4–11)	0.770
Vascular reconstruction, n (%)				0.032
None	59 (56)	40 (46)	99 (52)	
Venous	42 (40)	39 (45)	81 (42)	
Arterial	0 (0)	6 (7)	6 (3)	
Combined	4 (4)	2 (2)	6 (3)	
T stage, n (%)				0.492
T1	2 (2)	4 (5)	6 (3)	
T2	35 (33)	27 (31)	62 (32)	
T3	68 (65)	55 (63)	123 (64)	
T4	0 (0)	1 (1)	1 (1)	
N stage, n (%)				0.672
N0	20 (19)	17 (20)	37 (19)	
N1	66 (63)	50 (57)	116 (61)	
N2	19 (18)	20 (23)	39 (20)	
Lymph node ratio, median (range)	3 (0–29) / 27 (7–68)	3 (0–37) / 30 (10–103)	3 (0–37) / 28 (7–103)	0.303
Lymphagiosis carcinomatosa, n (%)	77 (73)	64 (74)	141 (73)	0.971
Venous invasion, n (%)	69 (66)	69 (79)	138 (72)	0.037
Perineural invasion, n (%)	95 (91)	78 (90)	173 (90)	0.384
Tumor differentiation, n (%)				0.678
G1	11 (10)	9 (10)	20 (9)	
G2	50 (48)	38 (44)	88 (46)	
G3	42 (40)	38 (44)	80 (42)	
G4	0 (0)	1 (1)	1 (1)	
Gx	2 (2)	1 (1)	3 (2)	
Tumor margins, n (%)				0.504
R1	34 (32)	32 (37)	65 (34)	
R0	71 (68)	55 (63)	126 (66)	
Length of ICU stay, days, median (range)	2 (0–53)	1 (1–16)	2 (0–53)	0.430
Length of hospital stay, days, median (range)	15 (3–83)	16 (2–60)	16 (2–83)	0.632
Readmission within 90 days, n (%)	13 (12)	13 (15)	26 (14)	0.601
90-day postoperative morbidity, n (%)	75 (71)	49 (56)	124 (65)	0.029
90-day major postoperative morbidity, n (%)	19 (18)	18 (21)	37 (19)	0.650
90-day postoperative mortality, n (%)	4 (4)	2 (2)	6 (3)	0.549
POPF, n (%) (total pancreaticoduodenectomy patients excluded)				0.947
None	72 (86)	46 (82)	118 (85)	
Biochemical Leak	5 (6)	4 (7)	9 (6)	
Type B	5 (6)	4 (7)	9 (6)	
Type C	2 (2)	2 (4)	4 (3)	
Reoperation, n (%)	14 (13)	14 (16)	28 (15)	0.590
Postpancreatectomy hemorrhage, n (%)	7 (7)	9 (10)	16 (8)	0.359
SSI superficial, n (%)	41 (39)	15 (17)	56 (29)	<0.001
SSI organ/space, n (%)	7 (7)	12 (14)	19 (10)	0.100
Pulmonary complication, n (%)	9 (9)	11 (13)	20 (10)	0.358
Cardiovascular complication, n (%)	12 (11)	8 (9)	20 (10)	0.614
Renal complication, n (%)	4 (4)	4 (5)	8 (4)	0.786
Neoadjuvant chemotherapy, n (%)	9 (9)	4 (5)	13 (7)	0.275
Adjuvant chemotherapy, n (%)	72 (69)	62 (71)	134 (70)	0.336
Adjuvant radiotherapy, n (%)	2 (2)	3 (3)	5 (3)	0.504

BMI, body mass index; ASA, American Society of Anesthesiologists; POPF, postoperative pancreatic fistula; SSI, surgical site infection.

**Table 2:** Comparison of clinicopathological characteristics of 148 patients undergoing partial/total pancreaticoduodenectomy for ductal adenocarcinoma of the pancreas according to utilization of ERCP/stenting based on the presence of severe or symptomatic jaundice or the need for neoadjuvant chemotherapy.

Variable	ERCP/Stenting (n = 61)	No ERCP/Stenting (n = 87)	All Patients (n = 148)	*p*-Value
Maximum level of bilirubin, µmol/L, median (range)	190 (12–592)	8 (2–646)	70 (2–646)	**<0.001**
Difference of bilirubin level before and after drainage, µmol/L, median (range)	101 (2–361)			
Intraoperative bile microbiology performed, n (%)	57 (93)	20 (23)	77 (52)	
Positive intraoperative bile microbiology, n (%)	57 (100)	7 (35)	64 (83)	<0.001
Length of ICU stay, days, median (range)	1 (0–17)	1 (1–16)	1 (0–17)	0.092
Length of hospital stay, days, median (range)	12 (3–70)	16 (2–60)	15 (2–70)	0.004
Readmission within 90 days, n (%)	9 (15)	13 (15)	22 (15)	0.985
90-day postoperative morbidity, n (%)	43 (71)	49 (56)	92 (62)	0.080
90-day major postoperative morbidity, n (%)	11 (18)	18 (21)	29 (20)	0.689
90-day postoperative mortality, n (%)	4 (7)	2 (2)	6 (4)	0.196
POPF, n (%) (total pancreaticoduodenectomy patients excluded)				0.667
None	43 (86)	46 (82)	89 (84)	
Biochemical Leak	4 (8)	4 (7)	8 (7)	
Type B	1 (2)	4 (7)	5 (5)	
Type C	2 (4)	2 (4)	4 (4)	
Reoperation, n (%)	7 (12)	14 (16)	21 (14)	0.428
Postpancreatectomy hemorrhage, n (%)	4 (7)	9 (10)	13 (9)	0.423
SSI superficial, n (%)	22 (36)	15 (17)	37 (25)	0.009
SSI organ/space, n (%)	3 (5)	12 (14)	15 (10)	0.078
Pulmonary complication, n (%)	6 (10)	11 (13)	17 (12)	0.598
Cardiovascular complication, n (%)	7 (12)	8 (9)	15 (10)	0.651
Renal complication, n (%)	4 (7)	4 (5)	8 (5)	0.604
Neoadjuvant chemotherapy, n (%)	9 (15)	4 (5)	13 (9)	0.032
Adjuvant chemotherapy, n (%)	49 (80)	62 (71)	111 (75)	0.456
Adjuvant radiotherapy, n (%)	1 (2)	3 (3)	4 (3)	0.504

POPF, postoperative pancreatic fistula; SSI, surgical site infection.

## Data Availability

The original contributions presented in this study are included in the article. Further inquiries can be directed to the corresponding author.
